# Reflections on Dry Eye Syndrome Treatment: Therapeutic Role of Blood Products

**DOI:** 10.3389/fmed.2018.00033

**Published:** 2018-02-23

**Authors:** Victor J. Drew, Ching-Li Tseng, Jerard Seghatchian, Thierry Burnouf

**Affiliations:** ^1^International PhD Program of Biomedical Engineering, College of Biomedical Engineering, Taipei Medical University, Taipei, Taiwan; ^2^College of Biomedical Engineering, Graduate Institute of Biomedical Materials and Tissue Engineering, Taipei Medical University, Taipei, Taiwan; ^3^Independent Researcher, London, United Kingdom

**Keywords:** dry eye syndrome, keratoconjunctivitis, artificial tears, serum eye drop, platelet lysate, blood

## Abstract

Dry eye syndrome (DES) is a multifactorial, frequent, pathology characterized by deficient tear production or increased evaporation of tears and associated with ocular surface alteration and inflammation. It mostly affects, but not exclusively, older individuals and leads to varying degrees of discomfort and decreased quality of life. Although the typical treatments of DES rely on using artificial tears, polyunsaturated fatty acids, integrin antagonists, anti-inflammatory agents, or on performing punctal occlusion, recently, standardized blood-derived serum eye drops (SED) are generating much interest as a new physiological treatment option. The scientific rationale in using SED for treating or releasing the symptoms of DES is thought to lie in its composition in multiple factors that resembles that of tears and contributes to the healing and protection of the ocular surface. This manuscript seeks to provide relevant background information on the management of DES, and on the increasing role that various types of SED or platelet lysates, from autologous or allogeneic origins, are playing in the improved therapeutic management of this pathology. The increasing role played by blood establishments in producing better-standardized SED is also addressed.

## Dry Eye Syndrome (DES): Epidemiology, Pathology, and Socio-Economic Impacts

### Epidemiology

Dry eye syndrome, also known as dry eye disease or keratoconjunctivitis sicca (KCS), is among the most common ocular complaints that older patients seek eye care for (1). Often under-recognized, DES is a multifactorial disease associated with varying degrees of discomfort and decreased quality of life (2). Awareness of DES varies much among populations, largely influenced by criteria used for self-diagnosis. For instance, in a survey conducted in Japan, 33% of participants estimated to be affected by DES (3). DES prevalence increases with age. One study found that there are no significant differences in DES prevalence in men of differing races or regions in the United States (1). Females of all age groups have a greater likelihood of developing DES than males, with DES prevalence increasing with age (4). Schaumberg et al. estimate that, in the United States, 1.68 million men over the age of 50 years experience DES and this number is expected to grow to 2.79 million by 2030 as life expectancy increases (1), whereas a previous health study found that over 3.23 million women are currently suffering from DES (4).

In another study, it was extrapolated that 4.3 million people over 65 years in the United States suffer from ocular irritation at least occasionally (5).

### Pathology

Dry eye syndrome pathology is typically divided into two types: deficient tear production or the evaporation of tears. Deficiency in tear production can be further divided into two more categories: Sjögren’s syndrome (SS), which is an autoimmune disease, or non-Sjögren’s syndrome (non-SS) (2, 6–8). Evaporation of tears refers to the loss of water from the ocular surface and is often the result of a meibomian gland dysfunction leading to a lipid bilayer deficiency in the tear film. The meibomian gland loses its function with age, leading to tear film instability and evaporation of tears; the quality and function of the meibomian gland has been linked, at least in part, to androgen levels (9). As males have higher androgen levels than females, this is consistent with the higher frequency of DES in females, especially after menopause.

Deficiency of tears caused by the decrease of aqueous tear production or excessive tear evaporation could increase osmolarity, with deleterious effects on the ocular surface. DES is associated with inflammation, and tear hyperosmolarity is an important mediator of this inflammation (2). Hyperosmolarity is associated with a key pathogenic mechanism of DES with negative effects on epithelial cells, including decreased cell volume, damage to DNA repair systems, increased apoptosis, and increased oxidative stress (2). It also stimulates multiple inflammatory events involving metalloproteinase-9 (MMP-9), tumor necrosis factor-alpha (TNF-α), and mitogen-activated protein kinase (MAPK) (2). Indeed, overexpression of proinflammatory cytokines/chemokines on the ocular surface has been found to be associated with the symptoms of dry eye (10–12), including interleukin (IL)-1β, IL-6, IL-17, IL-22, interferon-γ, tumor necrosis factor α (TNF-α), chemokine (C-Cmotif) ligand 2 (CCL2), and matrix metalloproteinases (13, 14).

In addition to dryness, symptoms associated with DES include pain, burning sensations, eye fatigue, redness, blurred vision, discharge, contact lens intolerance, sensitivity to light, and the feeling of foreign bodies present in the ocular region (2). Depending on the severity of DES, some patients experience problems carrying out basic daily activities such as reading, watching television, using a computer, driving a vehicle, and working (15). The discomfort caused by DES has also been tied to depression and decreased quality of life (2, 16–18). Furthermore, one study conducted a battery of tests, including tear function and ocular surface evaluations, and questionnaires on DES patients and determined a correlation between lower DES symptoms and patient happiness, suggesting that DES may influence a patient’s psychiatric well-being (19). A study of depression in DES subjects identified that these patients experience poor sleep quality (16). DES patients tend to sleep later, less, and use more sleep medications and antidepressants than non-affected subjects. However, antidepressants are being investigated as a potential contributor to DES (20), and patients with severe DES that progressively worsens over time suffer from increased anxiety and other mood disorders (2, 16).

### Socio-economic Impacts

Dry eye syndrome places a substantial economic burden on society due to hospital visits, medical costs, surgeries, and drugs, in addition to indirect costs such as loss of productivity (21). In the United States, the average DES patient makes approximately 6 hospital visits annually at a total cost of nearly $800 USD, adding up to a national cost of nearly $4 billion USD. These costs have risen over the years. When taking loss of productivity into account, annual societal costs are estimated to exceed $55 billion in the United States (22). In Europe, the estimated annual cost for ophthalmologist-managed care ranged from approximately $270 USD in France to $1,100 USD in the United Kingdom (21). In Japan, DES patient annual medical costs amounted to roughly $470 USD, mostly for drugs (21). Additionally, loss of work productivity in Japan was calculated to be approximately $536 USD per patient (21, 23). Surprisingly, the economic burden of DES due to loss of productivity drastically outweighed the direct expenses from receiving care from healthcare professionals or prescription drugs (22). Although the apparent costs vary among countries, the real costs of DES in each country are likely higher than data shows when taking into account that the purchase of over-the-counter artificial tears is not always incorporated into cost calculations (24) and data are incomplete, in particular in some parts of Asia (21).

## Current Therapeutic Strategies

The past 5 years have witnessed substantial developments in DES treatment options. Current treatment strategies that are not based on blood products include artificial tears, lubricants, steroids, immunosuppressant eye drops, dietary supplements associated with eyelid cleansing, and in more extreme case, anti-inflammatory drugs or punctal occlusion, a procedure consisting of inserting a plug into the tear drainage area to maintain tears in the eyes. Generally, such treatments, which can be combined, are selected based on disease severity and medical history of the patient. For the majority of DES cases, treatments focus on alleviating symptoms rather than addressing the causes of DES (6–8). Treatment effectiveness on symptoms must be regularly assessed (25). Regular use of artificial tears, anti-inflammatory drops, or punctal plugs provides only transient release and can often induce ocular side effects.

### Artificial Tears

The main functions of artificial tears are to increase moisture and provide lubrication of the ocular surface (26). There is a variety of artificial tear formulations available, differing in osmolarity, viscosity, electrolyte content, preservative content, and solute combinations (27). Artificial tears are currently formulated as osmoprotectants, with the purpose of restoring cell volume, decreasing cell stress, and reducing inflammatory reactions that occur under hyperosmotic conditions (28). One eye drop product uses propylene glycol (PG), polyethylene glycol (PEG), and hydroxypropyl guar (HP-Guar) with polyquaternium-1 preservative, which decreased ocular surface inflammation and DES symptom severity (29). Similarly, another eyedrop formulated using hyaluronic acid (HA) and trehalose stabilizes the bilipid membranes and protects labile proteins from desiccation, as well as prevents oxidative damage (30, 31).

A recent Cochrane analysis (27) could not identify whether different over-the-counter artificial tears provide “similar relief of signs and symptoms when compared with each other or placebo.” However, 0.2% polyacrylic acid-based artificial tears were found to be more effective than 1.4% polyvinyl alcohol-based artificial tears. In addition, artificial tears are not free of inducing some adverse events.

One limitation of artificial tears is the lack of some of the components of natural tears such as lipids, salts, proteins, and hydrocarbons, as well as growth factors, immunoglobulins, albumin, and vitamins present in serum, as discussed later (28, 32–34). Additional possible drawbacks of artificial tears include the presence of preservatives and other potentially toxic and allergenic compounds (35). Benzalkonium chloride (BAK), the most frequently used preservative compound in eye drops, may contribute to hyperosmolarity by disrupting tear films. BAK-induced damage extends to destruction of goblet cells, the corneal epithelium barrier, and deeper ocular tissues including release of proinflammatory cytokines, oxidative stress, and apoptosis (35). These factors should be taken into consideration when prescribing DES treatments.

### Polyunsaturated Fatty Acids (PUFAs)

Omega 3 and 6 fatty acids are essential fatty acids that cannot be synthesized in the human body. Their improper balance can lead to an omega 6 proinflammatory effect (8). Dietary supplementation of polyunsaturated fatty acids (PUFAs) may help manage DES (8, 36). In a randomized, double-blind study, omega-3 supplementation promoted tear film stabilization, reducing tear evaporation and DES symptoms as a result of increased goblet cell counts and improved epithelial cell morphology (8, 36). Balanced combination of omega-3 and omega-6 was recently found to attenuate contact lens-related DES (37).

### Integrin Antagonist

Lymphocyte function-associated antigen-1 (LFA-1), an integrin expressed on T-cells, is upregulated in the conjunctiva of DES patients (38). The interaction between LFA-1 and intercellular adhesion molecule-1 (ICAM-1) is key in T-cell adhesion with endothelial cells, as well as for T-cell interaction with antigen presenting cells (38). One approach to treat DES aimed to block the interaction between LFA-1 and ICAM-1. A small LFA-1 antagonist called Lifitegrast (SAR 1118) demonstrated in phase III clinical trials to significantly and safely relieve DES symptoms (39). Lifitegrast acts as an antagonist to LFA-1, resulting in the inhibition of T-cell activation, migration, and proliferation (40). However, other parameters to assess ocular function, such as Schirmer’s test results, tear breakup time, and inferior corneal staining, did not improve significantly (41). In July 2016, Xiidra^®^ was the first United States Food and Drug Administration (US-FDA)-approved LFA-1 agonist for treating DES (40).

### Anti-Inflammatory Therapies and Immunomodulators

Corticosteroids are one among several anti-inflammatory drugs to treat DES. In addition to reducing cellular infiltration, restoring vascular permeability and inhibiting chemotaxis, corticosteroids decrease fibroblast proliferation, reduce capillary dilation and suppress collagen deposition (40). They are considered highly effective toward the treatment of immune-mediated inflammatory diseases (40). However, their efficacy is limited to short-term usage (4 weeks or less) (41) as long term use leads to intraocular pressure and the formation of cataracts (40, 42). A combination with anti-inflammation agent (epigallocatechin gallate, EGCG) and mucoadhesive component, hyaluronic acid (HA) was used for the treatment of DES in a rabbit experimental model (43). Its therapeutic effect was evidenced via increased tear production, inflammation relief, and corneal epithelium recovery providing an alternative inflammatory inhibition agent for clinical DES treatment.

Cyclosporine is preferred over corticosteroids as a long-term treatment for DES. Cyclosporine A is a topical immunomodulator, first approved by the FDA in 2002 (Restasis^®^) for treating dry eye by increasing tear production (44) and by the European Union in 2015 (Ikervis^®^) (45). When administered topically, cyclosporine A acts as an immunomodulator, and when administered systemically, it acts as an immunosuppressant (8). This drug elicits anti-inflammatory properties by inhibiting cell-mediated reactions and preventing the release of proinflammatory cytokines, while upregulating the production of anti-inflammatory cytokines (44). Multiple studies have reported minimal side effects associated with topical application of cyclosporine A, under conditions increasing tear production and conjunctival goblet cell density (8, 45–47).

### Punctal Occlusion

Lacrimal punctal occlusion by plug is the most common non-pharmacological therapy for DES (48, 49). Although many authors recommend temporary occlusion by plugs as a trial treatment, permanent occlusion can be achieved through surgical obstruction of the lacrimal punctum. It has been described as being like “blocking the drain in a tub and collecting the water dripping from the tap” (50), which in other words means preventing tear drainage toward the nasal cavity by physically blocking the lacrimal punctum/canaliculus. Punctal occlusion is typically recommended for patients suffering from DES symptoms after failed attempts of using traditional aqueous treatment options (49). Although punctal occlusion may improve DES symptoms, there is a concern that it could retain unhealthy tears on the ocular surface causing irritation (51) and does not decrease tear cytokines and MMP-9 levels (52). An international panel of dry eye specialists recommended that factors associated with inflammation be handled prior to performing punctal occlusion (53). A study comparing the effects of administering punctal occlusion alone versus a punctal plug regime in combination with cyclosporine treatment demonstrated that for the near term, punctal occlusion, alone or with cyclosporine, yielded swift improvement in moisture. However, for the long term, treatment regimes involving punctal occlusion in combination with cyclosporine produced equal or superior results to treatment regimes using occlusion plugs only (50). A recent Cochrane study has identified a “very low-certainty evidence on symptomatic improvement” of punctal occlusion, commonly associated with epiphora and inflammatory conditions (54).

## Blood Product-Based DES Treatment Options

### Scientific Rationale

Human blood has been for many decades the source of a wide range of cell-based or protein-based therapeutic products. Cellular products include red blood cell (erythrocyte) concentrates, buffy coats/granulocytes concentrates, and platelet (thrombocyte) concentrates. Therapeutic proteins encompass coagulation factors, albumin, and immunoglobulins. More recently, new platelet-derived preparations, rich in growth factors, have been increasingly used for therapeutic applications in wound healing, tissue repair and regeneration (55), and *in vitro* clinical-grade cell propagation and tissue engineering (56).

There is now great interest in the application of human blood derived products as eye drops for DES. The most common blood product used as eye drops is serum, which is obtained by a physiological clotting process of blood collected without anticoagulant, as described in details below. The therapeutic benefits of blood-derived serum eye drops (SED) are probably multifactorial and may be explained by a composition that, in part, shares similarities with that of tears (32–34, 57). Like tears, SED contains carbohydrates, lipids, and various electrolytes, but 10 times more proteins including albumin, fibronectin, and transferrin (33). SED contains natural antimicrobial components, like complement component (58), and IgG, but less lysozyme than tears (32). Tears and SED provide vitamins and both share a similar osmolality (close to 300 mosm/l) as they contain comparable sodium and anion levels, and a similar pH (close to pH 7.4) (33, 59, 60). Potassium ion levels are about five times higher in tears than in SED, but calcium ions and phosphate levels are less in tears than in SED (33). However, the total protein content of tears is only about 10% that of SED (33). IgA is the major immunoglobulin in tears, playing a role in protecting against infections. Vitamin A is less in tears than in serum. Vitamin C and glutathione antioxidants are present at higher levels in tears than in serum. Most importantly, SED, like tears (61), also contain a mixture of cell growth promoting agents (62, 63), since blood clotting is associated with a degranulation of the platelets and a release of a plethora of growth factors from their alpha-granules (56, 64, 65). Growth factor composition is said to be qualitatively equivalent in tears and serum, but concentrations may be higher in serum, as is the case for transforming growth factor-beta (TGF-β) and platelet-derived growth factor (PDGF). Table 1 presents some of the known similarities existing between tears and SED.

**Table 1:** Comparison of tears and serum composition (individual variations may affect the values).

	Tears	Serum	Physiological function possibly relevant in ocular defect treatment
**Physico-chemical parameters** (33, 57)			
Osmolality, mosm/l	302	300	Maintains physiological osmolality and pH
pH	7.2–7.4	7.2–7.4	
**Proteins** (33, 55, 56, 74)			
Total proteins, mg/mL	7.37	60–70	Support tear surface tension, physiological hydration of the ocular surface, and ocular homeostasis
Albumin, mg/mL	0.05	35–40	Anti-apoptotic activity, detoxification
Fibronectin, μg/mL	21	200–300	Adhesion protein supporting wound healing
IgG, mg/mL	0.032	8–12	Anti-microbial
IgA, mg/mL	0.41		Anti-microbial
IgM, mg/mL	–	0.5	Endotoxin binding
IgD, μg/mL	–	3–300	
IgE, μg/mL	–	0.25–0.7	
Alpha 2-macroglobulin		2.6	Anti-collagenase
Complement system			Anti-microbial; bacteriostatic
Lactoferrin, mg/mL	1.51	–	Anti-microbial and anti-inflammatory
Transferrin, mg/mL	–	2–3	Iron-carrier; anti-microbial
Lysozyme, mg/mL	1.4	6	Iron carrier; anti-microbial
**Growth factors** (33, 55–57, 61)			
TGF-β1, ng/mL	2–10	6–50	Epithelial and stromal repair processes
PDGF, ng/mL	0.09–1.7	30–100	Enhances mitosis and scarring
EGF, ng/mL	0.2–3	0.5–1	Accelerates the migration of epithelial cells; anti-apoptotic
HGF, ng/mL	0.2–0.5	0.1–1	Supports corneal epithelial cells
VEGF, ng/mL	0.019	1–5	Supports conjunctival endothelial permeability
**Vitamins** (33)			
A, ng/mL	16–20	800–1000	Prevents squamous metaplasia and helps maintain the normal histology in the conjunctiva
C, μg/mL	117	7–20	Antioxidant
**Antioxidants** (33)			
Tyrosine, μM	45	77	
Glutathione, μM	107	ND	
**Electrolytes** (33)			
Na+, mEq/L	145	135–146	
K+, mEq/L	24.1	3.5–5.0	
Ca^2+^, mM	1.5	1.1	
Cl^−^, mM	128	96–108	
${\text{HCO}}_{3}^{-}$ mM	26	21–29	
${\text{NO}}_{3}^{-}$ mM	0.14	0.19	
${\text{PO}}_{4}^{3-}$ mM	0.22	1.42	
${\text{SO}}_{4}^{2-}$ mM	0.39	0.53	

### Serum Eye Drop

#### Preparation

Serum refers to the fluid portion of blood, devoid of cellular components that is obtained by letting blood collected without an anticoagulant to clot. It is typically prepared by collecting blood from patients (autologous source) or donors (allogeneic source), allowing the blood to clot for several hours prior to a centrifugation step at ca. 3,000 × *g* for approximately 10 min at 20–25°C to recover a supernatant serum. Serum may be passed through a 0.22-μm pore-sized filter for bacterial sterilization and clarification (34, 57, 66). In such a preparation, the platelets are not concentrated compared to the level found in the blood circulation, by contrast to newer SED formulation made from platelet concentrates where platelets are threefold to fivefold enriched compared to blood. When SED are made from platelet concentrates for transfusion, the content of serum plasma protein depends upon whether the platelets are suspended in 100% plasma or a mixture of plasma and platelet additive solution (PAS).

**Figure 1:**
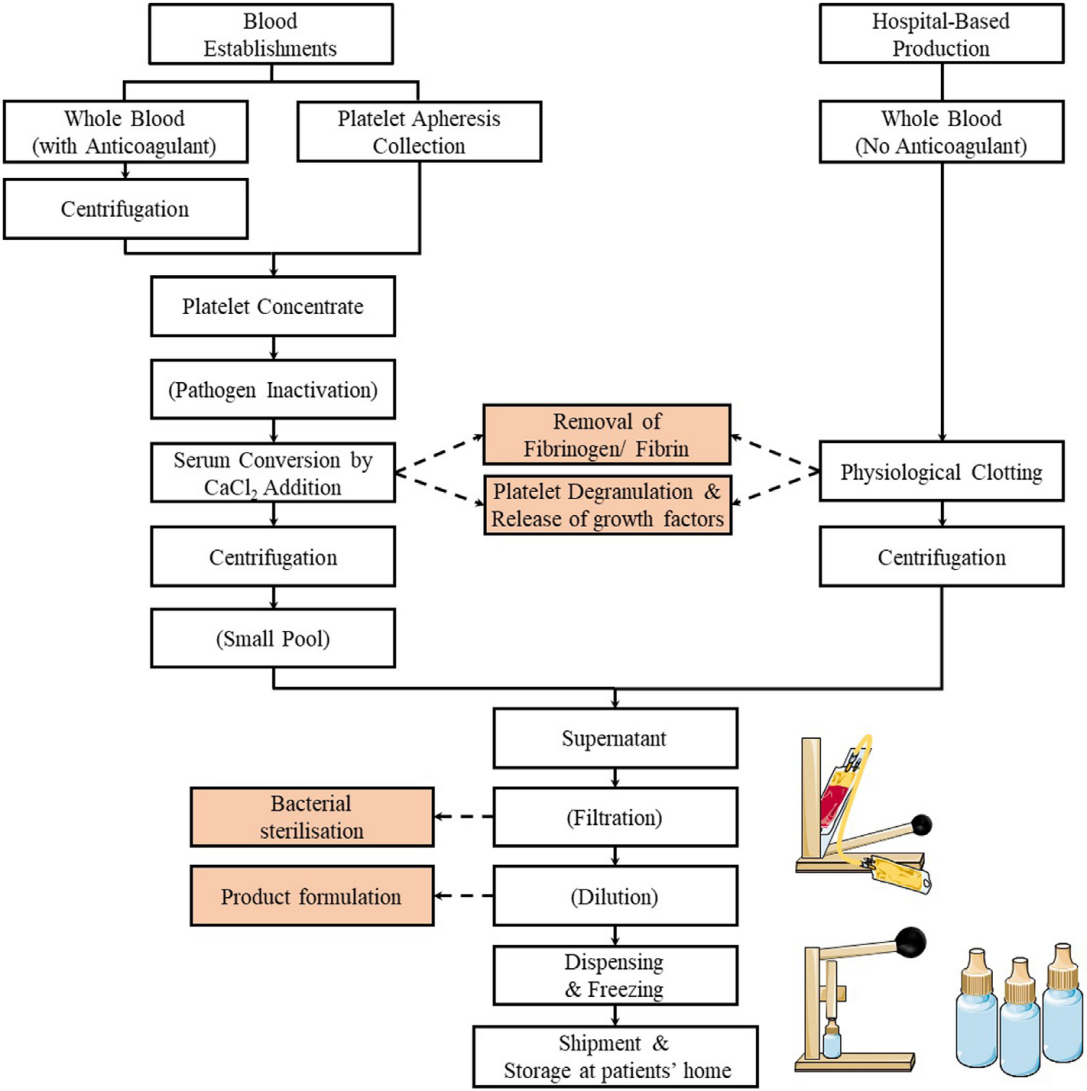
Flowchart of the preparation methods of serum eye drop by blood establishments and hospitals. Typically, the process involves the release of the platelet growth factors by physiological or CaCl_2_ stimulated clotting of blood (or platelet concentrates), followed by centrifugation, filtration (optional), dilution (optional), dispensing, and freezing.

An informative survey of methods used at international levels to prepare SED has recently been conducted by the Biomedical Excellence for Safer Transfusion (BEST) Collaborative (67). A summary of the preparation methods of SED is illustrated in Figure 1. Briefly, this survey indicates that SED for clinical use are prepared by national or regional blood establishments (also known as blood centers), as well as by hospitals or medical centers. Although most centers are manufacturing SED of autologous origin, an increasing number is now producing SED from allogeneic blood donors (68–70). When the SED are from allogeneic origins, procedures are in place, e.g., by preparing SED from AB group donors to hold a single blood group inventory or by donation screening to match all blood groups to ensure hemato-immunological matching between donors and recipients. It is, however, still unknown whether presence of anti-B agglutinins affect corneal healing (67).

A small majority of centers (most likely the blood establishments familiar with the production of blood components for transfusion) prepare SED from blood collected into blood bags rather than into tubes and use larger volumes of 200 mL or more. While the clotting time to get serum may be less than 6 h, it can be up to 24 h and (somewhat surprisingly) up to 3 days in some places. Most often, the serum is centrifuged to clarify the supernatant. Most centers do not perform a bacterial filtration step, whereas others do, implying that they apply the standard close-system manufacturing practices familiar to blood establishments. A small majority of the centers dilute the serum twofold to fivefold in saline or phosphate-buffered saline solution, before immediate dispensing in 0.5–5 mL aliquots into vials/eye dropper bottles or tubing segments before freezing (67).

#### Formulation

To date, the optimal formulation and dilution factor of SED for DES treatment remains uncertain. This is not unexpected considering the biological complexity of the serum material compared to artificial tears. Sometimes, the serum is diluted to approach the composition of tears and to decrease the concentration of TGF-β, which may exert an anti-proliferative activity and impair the healing of epithelial cells (33). There is, nevertheless, no real consensus yet nor evidence-based information on the optimal formulation (71). One cannot exclude that formulation may have to be adjusted to the disease treated or its extent (dryness or epithelium defect). Lower dilution factors (50%), or even no dilution at all, have been used (57, 72), while other authors have proposed to dilute SED to 20% in a sodium hyaluronate solution in particular to improve retention time and decrease the frequency of the administration (73). Higher SED concentrations have been reported to increase the speed of epithelial healing and closure in a patient recovering from laser *in situ* keratomileusis (LASIK) eye surgery (66).

#### Safety Aspects

Autologous SED do not essentially present risks of extraneous virus contamination when produced under GMP restricting the risks of cross-contamination or mislabeling with SED from another patient. Release testing focusing on microbial sterility of the final batch is carried out by about half of the producers that were recently surveyed (67). Preservative solutions are not added in SED; preparation procedures should therefore be carefully controlled and monitored to prevent bacterial contaminations.

Allogeneic blood donors donating blood for the production of SED should be screened for virus markers using the same standards that are applied to donations devoted to the manufacturer of transfused blood products (67, 74, 75). The main transfusion transmitted infections associated with allogeneic serum are viruses, most notably human immunodeficiency virus, and hepatitis B and C viruses (76). Emerging viruses, like West Nile virus, Dengue virus, Chikungunya virus, Ebola virus, and Zika virus, may also be a potential threat (77). However, efficient safety measures in place in blood establishments, namely donors’ screening and donation testing, dramatically restrict the risks of viral transmissions in a regulated blood collection jurisdiction (78). Particular future attention may need to address the pathological consequences of risks of transmission of other blood-borne viruses, such as the Herpes simplex virus, that may lead to ocular complications and affect vision (79).

Photochemical pathogen inactivation methods are in use for transfused plasma and platelet concentrates (80, 81), but they are not a current option as no dedicated or licensed pathogen inactivation treatment has been approved for application to therapeutic serum, although experimental studies have shown applicability to serum for cell expansion (82). As therapeutic platelet concentrates can be pathogen-inactivated using licensed treatment, this may speed-up the development of allogeneic pathogen-inactivated SED for clinical use (67). The well-established solvent-detergent (S/D) treatment, already applied to a wide range of biopharmaceutical preparations and plasma products (76), was experimentally proven applicable to rabbit SED (83). This S/D-treated rabbit serum was used as allogeneic SED equivalent to treat DES-rabbits, showing promising results. The safety and efficacy of such S/D-treated SED was demonstrated through the restoration of a corneal epithelium in a DES rabbit model. This preclinical study supports the possibility of using S/D virally inactivated SED to treat DES for the application of allogeneic human SED (83).

#### Shipment and Storage

Most often, patients themselves collect the SED from the production site and store the bottles at home in a domestic freezer. The typical specified shelf life set by producers of SED ranges from 3 to 12 months until thawing and up to 24 h to 1 week after thawing. Currently, SED storage at patients’ home is not specifically controlled and is under patients’ responsibility (33, 67). Studies have suggested that SED can be stored liquid at 4°C for up to 1 month, or frozen at −20°C or −80°C for up to 3 to 6 months, and in the dark to limit the decay in vitamin A (62, 71, 72). The stability of factors in serum, such as vitamin A, EGF, and TGF-β, was shown over up to 9 months. However, stability evaluations based on functional or biological activity (e.g., using cell cultures or animal models), rather than immunological tests (e.g., ELISA measurement of growth factors), should be conducted to determine the shelf-life. Furthermore, variations in the preparation methods of SED may impact its quality and properties (57, 72, 84) and, potentially, influence its long-term stability. Topical application of SED, which do not contain preservatives in order to prevent toxicity, requires careful handling to avoid microbial contamination.

#### Regulations

The regulatory status of current SED varies, but these preparations are typically regulated as blood products with variations from country to country depending upon jurisdictions (85). The increasing number of blood centers producing SED should eventually lead to the recognition and regulation of SED as a blood product, and to the establishments of international guidelines underlying their manufacture, and efforts towards implementing guidelines for standardization and product specifications. Clinical trials are expected to provide more rigorous information of clinical efficacy in various ocular pathologies and guidance for optimal products’ performance and clinical outcomes (67).

#### Clinical Rationale and Experience

The clinical strategy behind administering autologous serum is to take a comprehensive approach to treating dry eye, rather than just serve as a lubricant. Recent studies and review papers generally confirm the benefit of SED, from autologous or allogeneic sources, providing improved tear film stability, ocular surface health, and subjective comfort in refractory DES (57, 59, 71, 86–93). According to a Cochrane review based on a limited number of randomized clinical trials, autologous SED alleviate dry eye symptoms better than artificial eye drops for the first couple of weeks, but data still remain inconclusive at determining clinical efficacy over long-term periods (72). Therefore, randomized clinical trials involving larger cohorts of various patient groups should be conducted to better delineate the short-term and long-term benefit of SED in the treatment of DES and other ocular diseases (26, 72, 87).

#### Cost Consideration and Reimbursement Policy

Cost is a major limitation of using autologous SED. In the United States, most health insurance providers do not cover this form of dry eye treatment, resulting in out-of-pocket costs between $175 and $250 for a 2-month supply. The cost of this treatment may therefore makes it an option to consider for patients who have already exhausted more conventional forms of dry eye treatment.

#### Pending Issues

##### Autologous versus Allogeneic Products

Currently, there is no universal consensus of criteria on suitable patient selection for autologous blood donation (72). Another disadvantage of using autologous serum is that occasionally the frequent drawing of blood can be inconvenient to patients with prolonged treatment (59). For the elderly and for newborns with serious infections, autologous serum products may be unavailable or contraindicated (94). Cultural considerations are also playing some role. Patients of many Asian cultures, especially the elderly Chinese and Taiwanese, hold the belief that frequent venipuncture causes weakness and makes them more prone to bacterial infection (94). Also some people fear phlebotomy. Additionally, some patients may be too old to donate due to poor venous access or do not possess blood suitable for conversion into autologous SED due to clinical conditions such as previous cerebrovascular accidents, cardiovascular disease, anemia, use of anticoagulant medications or coagulation factor deficiency, or presence of inflammatory mediators (59, 70, 92). Allogeneic serum consists of the same general substances as those in autologous serum, but from a different source and provides a potential alternative treatment for these patients (59). Allogeneic SED are thus being researched for their efficacy in treating a variety of eye disorders associated with DES including persistent corneal epithelial defect (PED), KCS, chronic graft-versus-host disease (cGVHD), and many more (69, 88, 94). In other words, some ocular pathologies may actually benefit from SED made from allogeneic source, rather than autologous.

The use of allogeneic SED poses some risks of its own including the transmission of blood-borne pathogens, hypersensitivity and immune reactions, and potential legal or ethical concerns (59). To overcome some of the risks associated with allogeneic serum, some researchers have limited their investigations to SED obtained from family members (69). It has been reported that these eye drops are clinically comparable to autologous serum (94), but obtaining blood from family members does not imply the absence of risks, including infectious one’s. A ready-made, ABO-specific allogeneic eye drop study involving 34 patients (20 patients with KCS and 14 with PED) observed no side effects in any of the subjects and recorded objective improvement in 59% of the subjects. Of patients with KCS, relief was reported in 80% of the patients after allogeneic eye drop treatment (69). In a separate study investigating allogeneic serum in 36 PED patients, the epithelial defect of 16 subjects had healed in 2 weeks time (94). These results were confirmed with the observation of partial or full corneal changes in 16 of the 20 patients. This particular study supports the clinical potential for, and safety of, allogeneic eye drops. However, several immunological and physiological concerns still need to be given due consideration, namely ABO and HLA antibodies that may initiate inflammation (69). As such the virus safety and immune-hematological screening criteria of blood donations used to make allogeneic SED should be in line with those used for blood components for transfusion.

Due to the risk of transfusion-transmitted infections, it is highly recommended that manufacturers and documenters of allogeneic blood products implement good manufacturing practice as is recommended for the collection of blood components by blood establishments (67, 74, 75, 85).

##### Newer and Emerging Strategies Using Other Blood Products

Various other blood-derived preparations can be considered as therapeutic options to relieve DES symptoms and improve patients’ quality of life. It was identified, using a dry eye rat model, that plasma albumin provides a therapeutic benefit that was attributed to suppression of apoptosis (95). Albumin added to an eye drop formulation also helps to relieve DES symptoms in a rabbit model (96). Recent trends in development of blood products to treat DES focus on using blood fractions enriched in platelets (therefore equivalent to therefore somewhat equivalent to what is typically known as platelet-rich-plasma or PRP) as source material as the combination of platelet growth factors is believed to provide a scientific rationale to support its healing potential of DES (69). In the above-mentioned international survey (67), four centers manufacture eye drops either from (a) platelet-rich plasma (PRP) from human cords, (b) autologous platelet rich plasma donations, or (c) plasma. There is great interest in producing SED from PRP or platelet concentrate, as this blood fraction contains a threefold to fivefold higher platelet count than does whole blood.

A product termed Eye-PRP (“E-PRP”) is prepared by collecting whole blood in the presence of a 3.2% sodium citrate anticoagulant solution (97) in order to avoid serum formation. Anticoagulated whole blood is centrifuged to sediment red blood cells and to recover a platelet-enriched supernatant plasma. This PRP is then directly divided into aliquots of 3–4 mL and stored in 4°C refrigeration for 1 week or stored in −20°C freezer for extended periods (97, 98). Growth factors in E-PRP act to stimulate angiogenesis, promote cell repair, and activate macrophages (97). These essential molecules are actually commonly used in ophthalmology to promote epithelial wound healing of the cornea (98). 89% of patients using E-PRP eye drops four to six times per day reported subjective absence of DES symptoms. Benefits extended to include increased visual acuity, increased tear production, and improvements in ocular surface condition (98). A similar conclusion was reached by a study investigating the effect of this PRP on human lacrimal function (99).

An alternative to autologous serum and E-PRP is “plasma rich in growth factors” (PRGF). PRGF contains, like serum and E-PRP, a number of platelet growth factors, including platelet-derived growth factor, angiopoietin-1 (ANG-1), epidermal growth factor (EGF), VEGF, and many more (100, 101). PRGF can be prepared by collecting 30 mL of whole blood in tubes containing 3.8% sodium citrate and centrifuging the tubes, using soft spin, at 460 × *g* at room temperature for 8 min. The plasma supernatant portion is recovered, and the platelets are activated using 22.8 mM calcium chloride (102). Addition of calcium chloride induces a process of serum-conversion where a fibrin clot is generated, and growth factors are released due to platelet activation and degranulation. Afterward, the growth factor-rich supernatant serum is collected and filtered. It can be diluted with 0.9% sodium chloride down to 20%. All of these steps are performed under sterile conditions. The final product is distributed into eye drop dispensers, ready for use. For immediate usage, the eye drops could be stored in 4°C refrigeration up to 1 week, and for long-term storage, at −20°C for no longer than 3 months. Patients administer eye drop solution four times per day. Treatment cycles last approximately 3 months, but treatment can be extended several more months to include more cycles if symptoms do not improve. A study investigating the efficacy of PRGF to treat DES reported that out of 16 patients, 75% experienced moderate to substantial improvements. Use of PRGF has demonstrated an ability to reduce symptoms of squamous metaplasia in patients suffering from DES (101).

As mentioned above, the development of human platelet lysates (HPL) manufactured from platelet concentrates collected following the licensed procedures in place to prepare blood products for transfusion is very likely and opens the roadmap for the development of more standardized SED (103).

Finally, recently, a limited case study was conducted in the UK where patients applied a drop of whole blood to the affected eye(s) four times daily for 8 weeks. Significant improvements were noted in several parameters, such as visual acuity, corneal staining, tear break-up time (TBUT), and ocular comfort index (OCI), but not Schirmer’s test (104).

## Conclusion and Future Prospects

Dry eye syndrome is a common eye condition with a range of causes and degrees of severity and tremendous socioeconomic implications in addition to reductions in quality of life. There is a wide variety of medical products and procedures currently available or under development for the treatment of DES, each with their own advantages and disadvantages. Emerging treatment options include products derived from whole blood, such as autologous or allogeneic SED, E-PRP, PRGF, and HPL.

Relevant questions regarding the production method, quality, efficacy and safety of blood products used to treat DES remain, as already identified in particular with regards to standardization and formulation (87, 103). Similar to most claimed applications of platelet-derived preparations used in regenerative medicine, work is needed to design and standardize SED production methods to yield formulations with optimized blood proteins and growth factors composition to best address various DES and ocular pathologies. Reliable *in vitro* tests should be identified and validated as predictor of clinical outcomes (87). Furthermore, pre-clinical studies using valid animal models (105) to delineate the respective contribution of the plasma and platelet proteomes in the benefits of blood-derived eye drops in releasing DES symptoms should be performed. As is the case in other fields of regenerative medicine (106), dedicated platelet lysates may be needed to tackle the specific micro-environment of the diseased tissues and promote optimal repair strategies. An increasing involvement of blood establishments in producing SED is expected to contribute to improve standardization, quality, and safety (67).

In summary, blood products are well known for their benefits in relieving a variety of symptoms associated with DES. Many new and emerging blood products are currently being assessed for the presence of key growth factors and their overall effects weighed against their potential risks. Ultimately, as more evidence-based knowledge is obtained on the specific growth factors and their direct impact, patients with ocular defects should be able to receive personalized treatments, better customized to their individual needs and pathology, which are becoming the buzzword of all clinical interventions.

## Author Contributions

VJD and TB wrote the first draft. CLT made additions. JS reviewed and modified the final draft. All authors approved the final version.

## Conflict of Interest Statement

The authors declare that the research was conducted in the absence of any commercial or financial relationships that could be construed as a potential conflict of interest.
